# Heterotopic ovarian transplantation results in less apoptosis than orthotopic transplantation in a minipig model

**DOI:** 10.1186/s13048-016-0223-1

**Published:** 2016-03-15

**Authors:** Lia Cruz V. C. Damásio, José Maria Soares-Júnior, Jairo Iavelberg, Gustavo A. R. Maciel, Manuel de Jesus Simões, Ricardo dos Santos Simões, Eduardo Vieira da Motta, Maria Cândida Pinheiro Baracat, Edmund C. Baracat

**Affiliations:** Disciplina de Ginecologia, Departamento de Obstetrícia e Ginecologia, Hospital das Clínicas, Faculdade de Medicina da Universidade de São Paulo, São Paulo, Brazil; Instituto de Ensino e Pesquisa do Hospital Israelita Albert Einstein, São Paulo, Brazil

**Keywords:** Ovarian transplantation, Apoptosis, Pig, Cleaved caspase 3, Follicle number

## Abstract

**Background:**

Ovarian autotransplantation has shown increasing promise as a clinical method for the preservation of fertility and hormonal function. However, information regarding the success rate of this type of transplantation is limited. We hypothesized that results vary according to the site of the ovarian transplantation. To test this hypothesis, fresh or cryopreserved ovarian strips were autotransplanted to orthotopic or heterotopic sites. The strips were later collected, and the morphology and expression of selected markers of apoptosis were evaluated. We compared the Bax, Bcl-2 and cleaved caspase-3 staining levels and the morphometric aspects of autotransplanted fresh and cryopreserved ovarian strips placed at orthotopic and heterotopic sites in minipigs.

**Methods:**

Forty female minipigs were allocated to the following five groups: group 1 (control), ovarian tissue removed during oophorectomy; group 2, transplantation of fresh ovarian strips to a heterotopic site; group 3, transplantation of fresh ovarian strips to an orthotopic site; group 4, transplantation of cryopreserved ovarian strips to a heterotopic site; and group 5, transplantation of ovarian trips to an orthotopic site. On day 7 after transplantation, ovarian strips were collected, and the morphology and expression of apoptosis markers were evaluated.

**Results:**

In all groups, follicles across all stages of development were detected. The numbers of primordial, primary and secondary follicles were similar in all groups, but the numbers of antral follicles were lower in the cryopreserved groups in comparison with freshly derived ovarian tissue, with no significant differences observed between fresh and cryopreserved transplants. In all transplanted groups, Bcl-2 expression was lower and Bax expression was higher than in the control group. Furthermore, increased expression of apoptosis markers was detected in fresh intraperitoneal transplants. Lastly, the expression of cleaved caspase-3 was higher in the cryopreserved orthotopic group compared with the heterotopic group.

**Conclusions:**

Orthotopic and heterotopic ovarian strip transplantations are feasible options using these techniques. Importantly, we found that heterotopic transplantation preserves ovarian follicle integrity to a greater degree (i.e., lower expression of apoptosis markers) than orthotopic transplantation, and cryopreservation does not exacerbate expression of apoptosis’s markers. These findings have major clinical applications and enhance the discussion regarding the heterotopic transplantation of ovarian tissue.

## Background

Ovarian tissue transplantation can be used to preserve fertility and ovarian function in women experiencing premature ovarian failure and infertility [1]. An increasing number of women survive cancer every year; as a result, there has been an increased awareness of the impact of various cytotoxic treatments on gonadal function [2]. Among the current alternatives for fertility preservation, ovarian tissue transplantation differs from embryo and oocyte cryopreservation because it is the only procedure that can be offered to prepubescent girls and implemented without any delay in treatment [2–4]. This technique can be performed using fresh or cryopreserved tissue in an autologous or heterologous fashion at orthotopic or heterotopic sites [1].

In general, autologous ovarian transplants are more common than heterologous transplants due to potential problems related to immunosuppression. However, graft rejection through apoptosis or necrosis can occur even in autologous transplantation [4]. The mechanisms involved in apoptotic graft rejection remain largely unknown [1]. The few available studies of apoptosis in ovarian transplantation have suggested that this process participates in graft viability [5, 6]. Our study evaluated apoptosis in different implant sites using fresh or cryopreserved tissue.

Studies published on ovarian transplantation have been primarily experimental in nature and have involved a short evaluation period. Furthermore, these studies have been performed in small animal models using a range of differing techniques [1, 7, 8]. Although it is a relatively new technique, ovarian tissue transplantation is highly promising and requires further studies to standardize the techniques and to broaden clinical applications. Given that the minipig manifests macroscopic and physiologic characteristics similar to those observed in humans [9], this animal model was used to evaluate the viability of ovarian implants, either fresh or cryopreserved, following orthotopic or heterotopic transplantation. We hypothesized that the quality and viability of the transplanted tissue would differ according to the site of transplantation. Therefore, the objective of this study was to evaluate the tissue morphology and the expression of three markers of apoptosis (Bcl-2, Bax and caspase-3) in ovarian strips in non-cryopreserved ovaries (control) or after autotransplantation of non-cryopreserved or cryopreserved ovarian strips to orthotopic or heterotopic sites.

## Methods

### Experimental animals

Forty female pigs (*Sus scropha*) of the minipig br1 strain were used. The animals were between six months and one year of age, with a mean weight of 24.5 kg. Animals had experienced at least one estrous cycle in a farm setting prior to inclusion in our study to ensure that they were in the reproductive phase of development. The minipigs were maintained on a 12 h light–dark cycle and provided free access to water and food.

The present study was approved by the Ethics Committee for Research Project Analysis (CAPPesq) of the São Paulo University Medical School and by the Research and Teaching Israeli Institute (IIEP) of Albert Einstein Israeli Hospital.

After three days of acclimatization, each animal received synthetic progesterone (Regumate®) for 18 days for cycle synchronization, and after 5 days, the animals subsequently underwent the procedure according to experimental group assignment.

### Study groups

The animals were randomly divided into the following five groups (*n* = 8): GI, animals that only underwent bilateral laparoscopic oophorectomy, the ovarian tissue of which was removed during oophorectomy for use as the control; GII, animals that underwent bilateral oophorectomy and immediate autologous transplantation of fresh ovarian tissue into the subcutaneous tissue; GIII, animals that underwent bilateral oophorectomy and immediate autologous transplantation of fresh ovarian tissue into the peri-infundibular intraperitoneal site; GIV, animals that underwent bilateral oophorectomy and subsequent autologous transplantation of cryopreserved ovarian tissue into the subcutaneous tissue; and GV, animals that underwent bilateral oophorectomy and subsequent autologous transplantation of cryopreserved ovarian tissue into the peri-infundibular intraperitoneal ovarian region.

### Oophorectomy

For bilateral laparoscopic oophorectomy, the ovarian pedicle and utero-ovarian ligament were identified, clamped, cauterized and cut using a Stryker® endoscopy device. Ligatures were placed with a bipolar system for vessel sealing (Ligasure®).

### Preparation of ovarian fragments

The ovaries were collected, and the cortical region of each ovary was dissected from the adjacent tissue and medullary region in a sterile environment at room temperature using Leibovitz L-15 medium supplemented with 0.1 % bovine serum albumin (BSA). The cortical region was cut into 2-mm × 4-mm × 4-mm-thick fragments under sterile conditions. Ovarian cortical fragments were then freshly transplanted during the same surgery (GII and GIII) or subjected to a slow freezing process, stored for seven days in liquid nitrogen and then transplanted (GIV and GV). We used a heterotopic site in GII and GIV and an orthotopic site in GIII and GV for transplantation.

### Cryopreservation

The freezing protocol included a slow freezing process following a modified method described by Gosden et al. [10]. After oophorectomy, the intact ovary was transferred to a Petri dish that contained cold (4 °C) cryoprotectant (Leibovitz L-15). Ovarian strips were dissected from the adjacent tissue, and the cortex was dissected from the medullary region in Leibovitz L-15 medium containing 0.1 % BSA, NaCl fluid solution (10 %) and 0.1 mol/ml saccharose/mannitol. The fragments were placed in 1.2-ml cryotubes (cryogenic vials, Corning) with 1.5 M DMSO as a cryoprotectant. The cryotubes were capped, labeled with a cryogenic marker pen (Sigma-Aldrich; St. Louis, MO, USA) and placed in a programmable-rate freezer (Cryobath, CL-8800, Cryogenesis Software).

### Thawing

For thawing, the vials were removed from liquid nitrogen, placed at room temperature for 40 s and then immersed in a water bath at 37 °C for two minutes until the ice was melted. Ovarian cortical fragments were removed from the cryopreservation vials. The fragments were serially washed in phosphate buffered saline (PBS) supplemented with 1000 mg/l D-glucose and 36 mg/l pyruvate (Gibco) at room temperature and then stored in PBS until implantation [11].

### Subcutaneous transplantation

The groin fold was chosen as the subcutaneous region for autologous transplantation of ovarian tissue due to its easy surgical access. Furthermore, it is an inaccessible site for potential injury caused by the experimental animal or any other animal [12]. After the site was dissected, six ovarian tissue slices were inserted, the subdermal region was closed with a 3.0 VICRYL suture, and the skin was closed with a 3.0 nylon suture. After dissection and preparation of the strips, the mean time required for subcutaneous reimplantation was 2.2 min.

### Intraperitoneal transplant

The pelvic cavity was accessed by laparoscopy. The intraperitoneal site used was the parietal peritoneal fold near the uterine tubes. Six ovarian tissue strips were initially fixed to a Surgicel® mesh with 4.0 VICRYL sutures and subsequently inserted into the abdominal cavity through an 11-mm trocar (Ethicon). The ovarian strips were sutured to the parietal peritoneum fold, near the left uterine tube, with intracorporeal 3.0 VICRYL sutures. After dissection and preparation of the strips, the mean time required for intraperitoneal reimplantation was 5.8 min.

### Tissue collection and histological evaluation

The ovarian strips were collected 30 days after autotransplantation in GII and GIII (fresh) and 37 days after autotransplantation in GIV and GV (cryopreserved), with a 7 day interval for post-operatory recuperation and another administration of synthetic progesterone (Regumate®) for 18 days for cycle synchronization; then, after 5 days, the animals subsequently underwent the procedure according to experimental group assignment. Samples were fixed in formalin, embedded in paraffin, and cut into 5-μm sections for histological preparation. These tissue samples were then transferred to a 70 % ethanol solution and stored at room temperature until processing. A detailed histological description of the ovarian tissue was prepared for each animal and characterized for each experimental group according to aspects of the germinative epithelium, cortex and stroma.

Hematoxylin- and eosin-stained samples on glass slides were evaluated for the quality and density of the follicles in the ovarian tissue implants and for the general aspects of tissue organization and vascularization. Follicle counting and classification were performed according to Israely et al. [13] and Maciel et al. [14]. All tissue pieces were evaluated.

Ovarian follicles were classified into the following six categories: primordial follicles, primary, antral healthy follicles, atretic follicles, the corpus luteum and the corpus albicans. Then, the number of each follicle type was determined per tissue area.

A primordial follicle was defined as a follicle that exhibited only one oocyte and a single layer of flat squamous cells [14–16]. Preantral growing follicles developed from the primordial follicles but exhibited no antrum formation. The preantral follicles were classified into the following groups: the primary follicle, with one oocyte and a single layer of cuboidal squamous cells; the secondary follicle, with one oocyte and two to eight layers of granulosa cells and no antral cavity; and the tertiary (antral) follicle, with more than eight layers of granulosa cells and no antral cavity [14, 17]. Follicles with an antral cavity, regardless of their size, were considered to be antral follicles [14]. In all these cases, an oocyte had to be visible for a follicle to be classified. The corpus luteum presented typical luteal cells with large nuclei and vessels in the periphery [15, 18]. The corpus albicans was described as amorphous hyaline tissue resulting from corpus luteum regression [19].

The follicle counts were performed on all slides with an 8-key Manual Volume Counter (Digitimer model) with a totalizer. The count was confirmed in random samples obtained from each group using a computerized image analysis program. Image capture and measurements were performed using a light microscope (Carl Zeiss) coupled to a high-resolution camera (AxioCam MRc, Carl Zeiss) and color video monitor. The AxioVision Rel 4.6 program (Carl Zeiss) was used for image analysis.

### Immunohistochemical analysis

Deparaffinization and washing were performed using standard histochemical techniques. The tissue sections were incubated at 4 °C overnight in a dark chamber with anti-Bcl-2 (monoclonal), anti-Bax (polyclonal) or anti-cleaved caspase-3 (polyclonal) (IMUNY Biotechnology, Brazil) primary antibodies diluted to 1:400 in PBS. After the tissue sections were repeatedly washed in TBS containing 0.1 % Tween 20, primary antibodies were detected using biotinylated mouse anti-IgG secondary antibody at 1:400 for the anti-Bcl-2 antibody or with biotinylated rabbit anti-IgG antibody at 1:400 for the anti-Bax and anti-cleaved caspase-3 antibodies. Subsequently, the samples were incubated with AB reagent (avidin-biotin complex) for 30 min at room temperature (Santa Cruz Biotechnology Inc., Santa Cruz, CA, USA) and developed with DAB (Sigma Chemical Co.; Saint Louis, MO, USA) for five minutes at room temperature. The sections were then counterstained with hematoxylin solution.

The above apoptotic markers were evaluated using an optical microscope with 10× ocular and 40× objective lenses (Nikon Eclipse E200). The relative quantity of cell staining per area was analyzed by visualizing ten distinct fields in each slide. Ten slides per animal were assessed. Immunohistochemical data were evaluated using Image Pro Plus image capture software, and ImageJ Plus software was used to analyze the number of follicles, the follicles per tissue area and the intensity of staining of the images.

### Statistical analysis

The histomorphological and immunohistochemical intensity data were expressed as the mean and standard deviation. The Kruskal-Wallis test and Dunn’s post-test were used to evaluate the histomorphometrical differences between the groups, including the number of follicles, the follicles per tissue area, the intensity of staining and the expression of markers of apoptosis. ANOVA was used to analyze the expression of Bcl-2, Bax and cleaved caspase-3. Tukey’s test was used for intergroup transversal evaluation. A significance level of *p* < 0.05 was set. The data were analyzed using GraphPad Prism software, version 5.0.

## Results and discussion

### Histomorphometric analysis

**Fig. 1:**
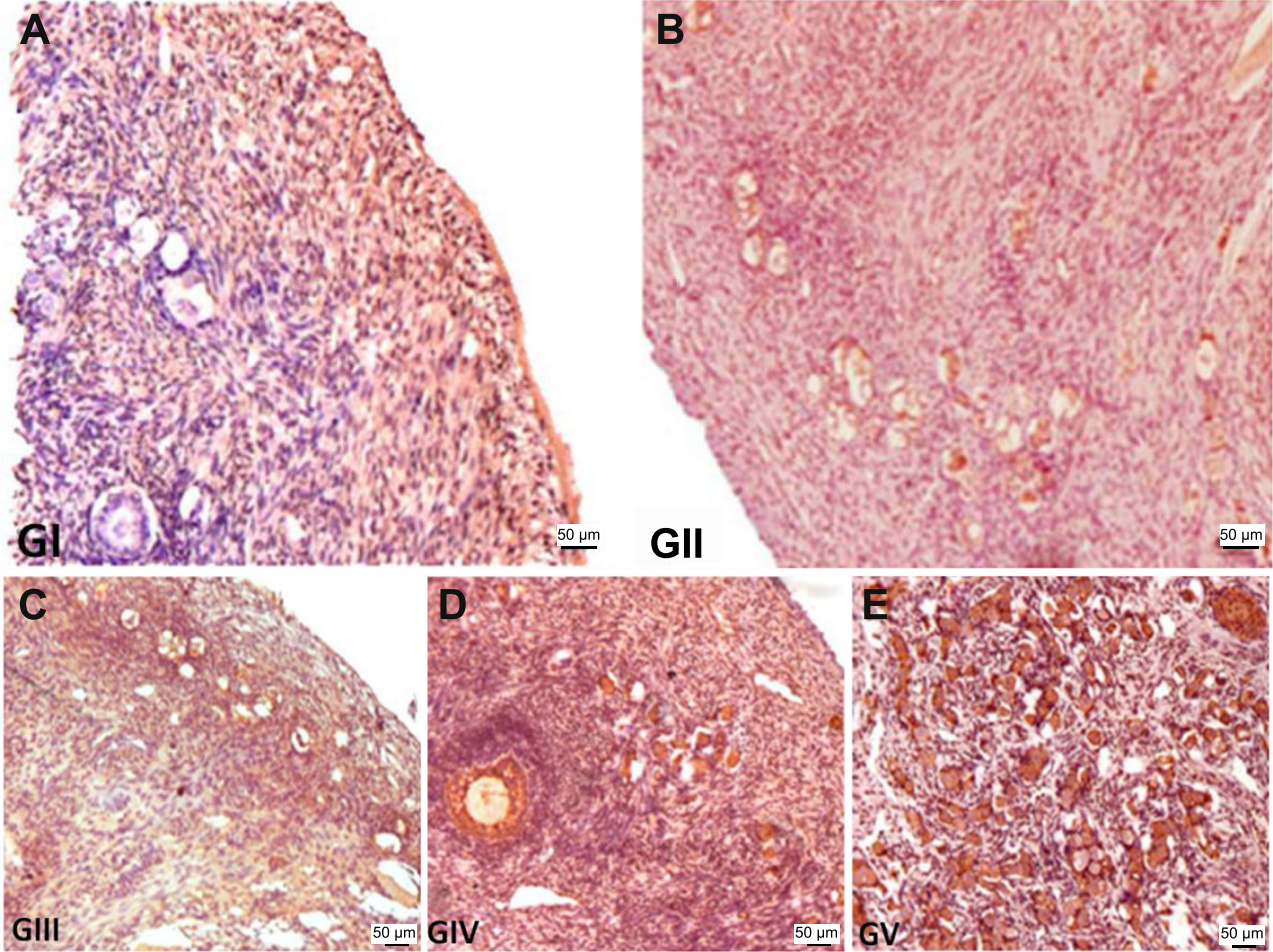
Photomicrographs of histological sections of minipig ovaries. Follicles in all stages of development was detected across all groups (bar = 50 μm; HE staining, 100×). **a** - animals that only underwent bilateral laparoscopic oophorectomy, the ovarian tissue of which was removed during oophorectomy for use as the control (GI); **b** - animals that underwent bilateral oophorectomy and immediate autologous transplantation of fresh ovarian tissue into the subcutaneous tissue (GII); **c** - animals that underwent bilateral oophorectomy and immediate autologous transplantation of fresh ovarian tissue into the peri-infundibular intraperitoneal site (GIII); **d** - animals that underwent bilateral oophorectomy and subsequent autologous transplantation of cryopreserved ovarian tissue into the subcutaneous tissue (GIV); **e** - animals that underwent bilateral oophorectomy and subsequent autologous transplantation of cryopreserved ovarian tissue into the peri-infundibular intraperitoneal ovarian region (GV)

In our control sample (GI), the ovary was covered with simple cuboidal or columnar epithelium and formed by well-defined medullary and cortical regions that exhibited numerous ovarian follicles across all stages of development. The morphology of the ovarian strips in the heterotopic transplantation groups (GII and GIV) was similar to that of the control group. In all transplanted groups, a lower concentration of primordial follicles and greater amount of connective tissue area were observed, especially in the orthotopic transplantation groups (GIII and GV, Fig. 1).

A global follicle count with corresponding distributions was performed in all groups, and the data are summarized in Table 1.

**Table 1:** Histomorphometry of pig ovaries (minipig) in the different study groups

	GI	GII	GIII	GIV	GV
Primordial	388.17 ± 508.53	440.88 ± 431.28	442.21 ± 49.80	376.81 ± 164.78	317.63 ± 168.66
Primary	19.50 ± 15.46	18.38 ± 13.64	16.74 ± 2.68	14.40 ± 6.11	11.99 ± 6.22
Secondary	10.33 ± 18.53	12.75 ± 13.29	13.73 ± 3.45	12.01 ± 4.97	10.03 ± 4.57
Tertiary	3.25 ± 3.30	4.38 ± 3.66	3.65 ± 0.52	3.13 ± 1.34	2.78 ± 1.51
NC^a^	18.17 ± 25.39	27.25 ± 29.43	25.06 ± 4.88	21.70 ± 9.06	19.56 ± 10.17
Healthy Antral	3.00 ± 0.82	1.75 ± 1.49	1.76 ± 0.91	1.62 ± 0.79*	1.39 ± 0.43*
Atretic Antral	11.67 ± 5.43	8.13 ± 4.94	7.54 ± 3.09	6.80 ± 3.00	5.58 ± 2.24
Corpora lutea	4.67 ± 2.34	2.88 ± 2.80	3.17 ± 1.03	2.81 ± 1.18	2.31 ± 0.95
Corpora Albicantia	1.67 ± 1.97	1.88 ± 2.17	1.92 ± 0.21	1.63 ± 0.72	1.42 ± 0.78
Total	458.17 ± 567.20	516.88 ± 478.49	505.18 ± 47.98	428.98 ± 190.32	361.30 ± 195.26

Data shown are the means ± standard deviations

^a^ *NC* Not classified- the microscopic aspect of the development follicle was insufficient to classification

*Indicates a significant difference with a *p*-value = 0048 and *p*-value = 0031, GIV and GV, respectively, compared to GI

### Immunohistochemical analysis

**Fig. 2:**
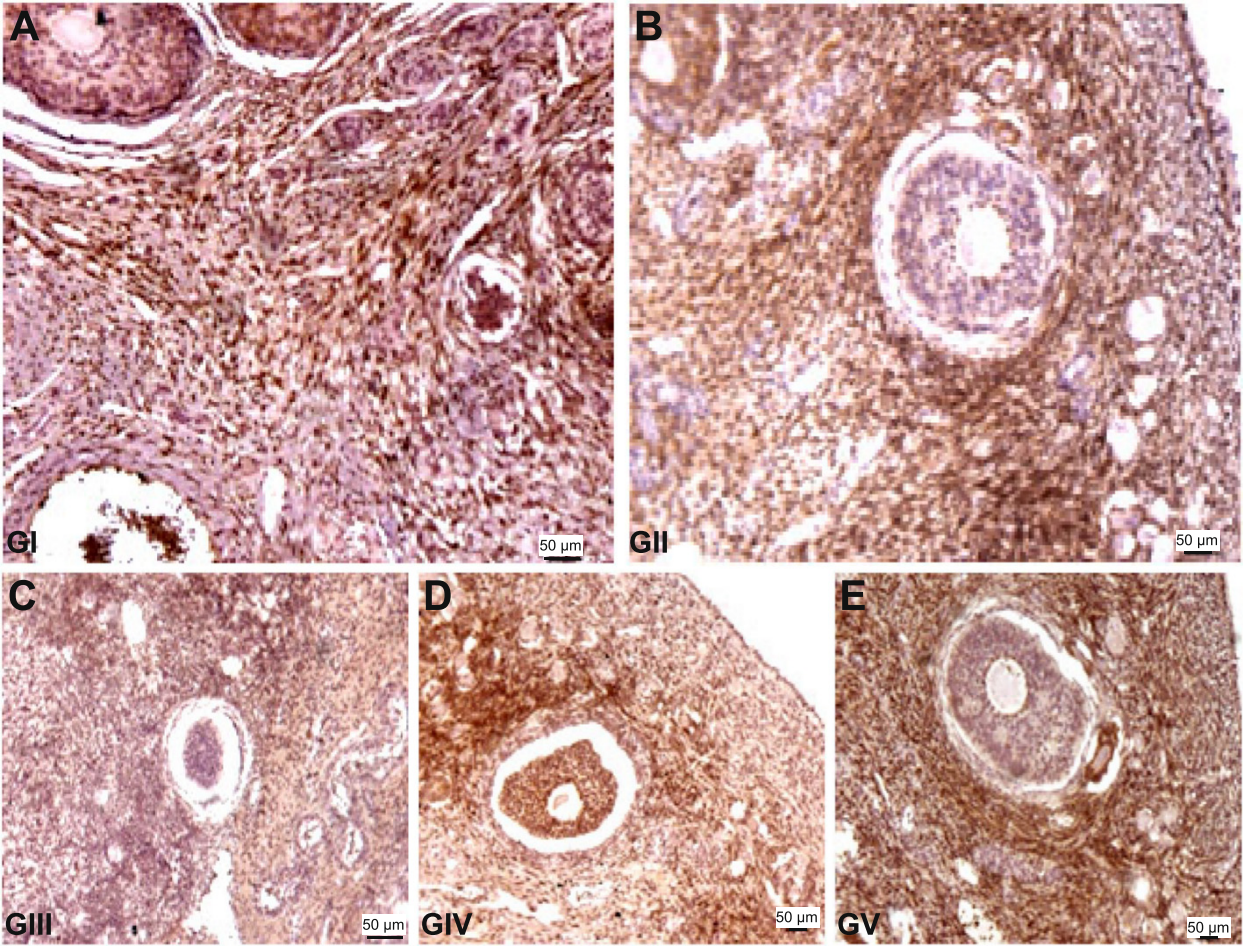
Photomicrographs of histologic sections from minipig ovaries. Higher expression of cleaved caspase-3 was detected in follicle cells in GV (bar = 50 μm; immunohistochemistry, 100×). **a** - animals that only underwent bilateral laparoscopic oophorectomy, the ovarian tissue of which was removed during oophorectomy for use as the control (GI); **b** - animals that underwent bilateral oophorectomy and immediate autologous transplantation of fresh ovarian tissue into the subcutaneous tissue (GII); **c** - animals that underwent bilateral oophorectomy and immediate autologous transplantation of fresh ovarian tissue into the peri-infundibular intraperitoneal site (GIII); **d** - animals that underwent bilateral oophorectomy and subsequent autologous transplantation of cryopreserved ovarian tissue into the subcutaneous tissue (GIV); **e** - animals that underwent bilateral oophorectomy and subsequent autologous transplantation of cryopreserved ovarian tissue into the peri-infundibular intraperitoneal ovarian region (GV)

Follicles in the transplantation groups, notably GIII, exhibited weaker Bcl-2 staining. In addition, Bax expression was more frequent and more intense in the follicles of the transplant groups than in those of the control group (GI). This observation was most evident in the orthotopically transplanted fresh ovary group (GIII, Fig. 2).

We observed no cleaved caspase-3 staining in the stromal follicular cells in GI, although there was light staining in the corpus luteum. In GII, the stroma also showed no staining, and the theca follicular cells were moderately stained. The general GII aspect was similar to that of GI, except for greater staining in some areas of different corpus lutea specimens. In GIII, the follicles were moderately stained, and staining also appearing in the theca interna; in contrast, the stroma showed weak staining. In GIV, we observed stained and unstained follicles, and although all clusters and stroma were lightly or moderately stained, the staining was weaker than that observed in GIII. In GV, the presence of stained and unstained follicles and lightly or moderately stained clusters and stroma (similar to those observed in GIV) were detected.

The immunoexpression of Bcl-2, Bax and cleaved caspase-3 and the Bcl-2/Bax ratio were quantitatively evaluated using Image J Plus software based on pixel intensity regarding the follicle type. These results are detailed in Table 2.

**Table 2:** Distribution of Bcl-2, Bax and cleaved caspase-3 expression in the different study groups

	GI	GII	GIII	GIV	GV
Bcl-2	143.24 ± 23.77^a^	68.36 ± 12.05	40.66 ± 23.96	49.47 ± 13.56	50.93 ± 12.53
Bax	75.69 ± 24.80	97.18 ± 34.22^b^	125.30 ± 15.68^c^	107.79 ± 20.09	97.98 ± 24.98
Bcl-2/Bax	2.17 ± 1.11^d^	0.77 ± 0.27	0.32 ± 0.17	0.38 ± 0.22	0.45 ± 0.32
Cleaved Caspase-3	121.1 ± 25.50	159.30 ± 26.1	151.0 ± 28.9	135.0 ± 22.30	170.09 ± 23.60^e^

Data shown are the means ± standard deviations

^a^Indicates a significant difference with a *p*-value less than 0.05 compared with GII (*p* = 0042), GIII (*p* = 0039), GIV (*p* = 0038) and GV (*p* = 0037)

^b, c^Indicates a significant difference with a *p*-value less than 0.05 compared with GI (*p* = 0049 and *p* = 0021, respectively GII and GIII)

^d^Indicates a significant difference with a *p*-value less than 0.05 compared with GI. (*p* = 0032), GIII (*p* = 0013), GIV (*p* = 0022) and GV (*p* = 0027)

^e^Indicates a significant difference with a *p*-value = 0045 compared with GI

The minipig (*Sus scropha*) is an experimental model that provides advantages over rodents and small vertebrates based on its anatomical and physiological resemblance to humans [9]. The female pig and human reproductive systems share many similarities, including ovary size and density (follicles per tissue area), resistance to manipulation of the ovarian cortex and the length of the estrous cycle and the surgical techniques used for implantation [20]. However, female pigs are polyovulatory and respond readily to ovarian stimulation, which is not typical of humans [21].

The development of minipigs that are not carriers of nanism has circumvented the problem of using conventional strains. Even as adults, minipigs are sufficiently small to use as laboratory animals in biomedical research [9]. In our study, the minipig served as an appropriate model for laparoscopic surgeries in which to evaluate ovarian tissue transplantation.

There are two primary approaches for autotransplantation of ovarian tissue: heterotopic, in which cortical fragments can be transplanted subcutaneously or to other sites; and orthotopic, in which the fragments are transplanted into their original site(s) or into the remaining ovary near the ovarian fossa or infundibulopelvic ligaments [2]. A procedure using cortical fragments without the vascular pedicle yields more full-term pregnancies in humans after autologous transplantation compared with the use of the whole ovary [4]. Heterotopic transplantation has some advantages over orthotopic transplantation, as the former is a simpler technique and permits superior access for oocyte retrieval and the monitoring of neoplasms [22]. The disadvantages of heterotopic transplantation include cosmetic concerns, potential adverse effects on oocytes conferred by an environment different from the intra-abdominal pelvic region and the presumed need for assisted reproduction to achieve fertility [22]. In our study, ovarian transplantation using an animal model resulted in satisfactory ovarian function as reflected by follicle count for both the subcutaneous orthotopic and heterotopic implants located near the pelvic infundibulum.

Primordial follicles are theoretically more resistant to cryoinjury given that each primordial follicle contains a small oocyte with a relatively quiescent metabolism and no fragile meiotic spindles, zona pellucida or cortical granulations. Collectively, these factors facilitate the penetration and action of the cryoprotectant [4]. However, apoptosis has been observed in cryopreserved and transplanted primordial follicles, especially in those primordial follicles transplanted orthotopically. This study illustrates that fresh transplantation is superior to transplanting cryopreserved tissue.

Harrison performed ovarian transplants in pigs and observed functional ovarian reactivation manifested by estrus cycles and the occurrence of full-term pregnancies [21]. Our study differed from this study in that we transplanted the ovarian tissue into two different sites to determine whether the subcutaneous tissue provided advantages over the peritoneal tissue in terms of apoptosis.

Experimental studies of ovarian transplantation in sheep have reported that fertility can be restored upon transplantation of cryopreserved ovarian cortical strips [10, 23]. Another important study established the feasibility of the autologous transplantation of ovarian tissue into the dorsal subcutaneous tissue in rats, with rates of endocrine function preservation greater than 80 % [7]. However, unlike our study, these prior efforts did not assess apoptosis in the transplanted tissues. Apoptosis is the central mechanism of cell death in the ovary and a fundamental marker for the assessment of transplant success [24, 25]; however, apoptosis has not been well studied in regards to ovarian transplantation [5, 6, 26].

We sought to determine differences in morphologic features, follicle count and distribution and evidence of apoptosis in the implants. We found a superior response in heterotopic implants compared with orthotopic implants, including the observation that the heterotopic cryopreserved implants lost fewer follicles in comparison to orthotopic implants. Our findings are consistent with those of other reports [1] and suggest that fresh and subcutaneous transplants exhibit a potential that has thus far not been effectively utilized in research [8, 27, 28].

Across the groups, follicles in all stages of development, pre-ovulatory follicles and corpus lutea were similar in number and morphology to those observed in the control group. These findings suggest that the implant’s physiological functions and responses were intact, and these data are in agreement with the current concept that ischemia may have more influence on follicle loss than the process of cryopreservation prior to transplantation [3, 8, 27].

The more significant results of apoptosis in intraperitoneal transplants may be attributed to structural changes and follicle loss that are secondary to lengthy ischemia after tissue removal. Alternately, these changes may reflect exposure to room temperature during experimental manipulations during the preparation and fixation of the strip for transplantation into the intraperitoneal region [3, 17].

Importantly, these transplants were autotransplants with fresh tissue immediately transplanted at the time of oophorectomy, whereas cryopreserved tissue was transplanted 7 days after oophorectomy. Because the ovarian cycle of these animals was synchronized before oophorectomy, the tissues are synchronized between the transplanted groups; however, the animals’ gonadotropin levels were likely very different (higher in the cryopreserved recipient group, which could lead to better antral follicle numbers), which could be a confounding factor in our study design. Unlike what was expected, our results did not show a statistically significant increase in the number of atretic follicles or a statistically significant decrease in healthy antral follicles, and in addition to indicating a confounding factor, these results may denote an influence of the previous hormonal stimulation.

Laparoscopic transplantation is a lengthier procedure and involves more manipulation and trauma to tissue strips than the subcutaneous transplantation performed in this study. This procedure, laparoscopic transplantation, is the most widely used technique for oophorectomy and the reported cases of orthotopic ovarian transplantation in women [2]. However, there have been no studies comparing orthotopic and heterotopic implants in the minipig model.

Massardier et al. described a laparoscopic technique for orthotopic transplantation using fresh and cryopreserved tissue in sheep; however, the group did not observe significant differences in the total follicle count [27]. Our study agrees with this previous study and contributes new information to the topic by comparing orthotopic and heterotopic transplantation and by immunohistochemically evaluating apoptosis.

## Conclusions

Our data suggest that fresh ovarian tissue transplanted into a subcutaneous site exhibits a lower rate of apoptosis; therefore, this transplantation approach is favorable for preserving ovarian viability. The potential clinical implications involve the use of heterotopic sites for ovarian transplantation in humans. These findings have extensive implications for the clinical application of ovarian transplantation techniques and will enhance the discussion about the heterotopic transplantation of ovarian tissue. The possible methodological advantages in humans include improved tissue monitoring, potential use in a pelvis not suitable for transplantation and easier access for additional fertility procedures. The data were consistent with a superior response but it is an experimental study and further studies are needed.
